# Microglial Modulation as a Therapeutic Avenue for Perioperative Neurocognitive Disorders: Unveiling Pathophysiological Mechanisms and Clinical Implications

**DOI:** 10.1111/cns.70481

**Published:** 2025-07-18

**Authors:** Xizhen Liu, Anke Zhang

**Affiliations:** ^1^ Department of Anesthesiology The Affiliated People's Hospital of Ningbo University Ningbo China; ^2^ Department of Neurosurgery The Second Affiliated Hospital, School of Medicine, Zhejiang University Hangzhou China

**Keywords:** cognitive decline, microglia, neuroinflammation, perioperative neurocognitive disorders, surgical stress, therapeutic strategies

## Abstract

**Background:**

Perioperative neurocognitive disorders (PND) encompass a spectrum of cognitive impairments that arise from the preoperative phase and can persist for months after surgery, with a prevalence of up to 50% in elderly patients. These disorders, including postoperative delirium and sustained cognitive decline, significantly reduce patient quality of life and impose substantial burdens on healthcare systems. Despite extensive research, the underlying pathophysiological mechanisms remain inadequately understood, limiting the development of effective treatments. Increasing evidence highlights neuroinflammation as a central factor in PND, with microglia—the resident immune cells of the central nervous system—playing a key role in mediating inflammatory responses that lead to cognitive dysfunction.

**Methods:**

This review comprehensively analyzes the role of microglia in the pathogenesis of PND. It details key perioperative triggers of microglial activation, such as surgical stress, anesthesia, and systemic inflammation. The review further examines preclinical interventions aimed at modulating microglial function, including depletion strategies, polarization toward anti‐inflammatory phenotypes, and inhibition of inflammatory pathways like NF‐κB and NLRP3.

**Results:**

Preclinical studies demonstrate that dysregulated microglial activation results in excessive production of pro‐inflammatory cytokines, oxidative stress, and synaptic disruptions, which collectively contribute to neuronal injury and cognitive impairment. Interventions targeting microglial activation have shown efficacy in reducing neuroinflammation and preserving cognitive function in animal models.

**Conclusions:**

Targeting microglial activation represents a promising strategy for alleviating PND. However, challenges remain in clinical translation, necessitating advanced drug delivery platforms, personalized therapeutic approaches, and rigorous clinical validation. Advances in microglial modulation hold potential for improving postoperative cognitive outcomes and enhancing patient recovery.

## Graphical abstract

Microglia interact with oxidative stress, reactive astrocytes, neuronal damage, neuroinflammation, synaptic dysfunction, mitochondrial dysfunction, epigenetic modifications, and blood–brain barrier disruption, playing a critical role in the pathophysiology of perioperative neurocognitive disorders (PND). Targeting microglia presents a promising therapeutic strategy for the management of PND.

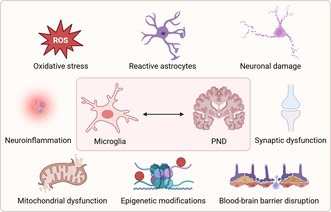

## 1 Introduction

Perioperative neurocognitive disorders (PND) encompass a spectrum of cognitive impairments that arise during the perioperative period, spanning from the preoperative phase to months post‐surgery [1, 2]. These disorders, including postoperative delirium, delayed neurocognitive recovery, and persistent postoperative neurocognitive dysfunction, are increasingly recognized as significant complications, particularly among the elderly, where prevalence rates can reach 50% [3]. The high incidence of PND not only compromises patient quality of life but also imposes substantial burdens on healthcare systems by prolonging hospital stays, increasing morbidity, and escalating medical costs.

Despite extensive research, the pathophysiological mechanisms underlying PND remain inadequately defined, impeding the development of effective preventive and therapeutic strategies. Growing evidence implicates neuroinflammation as a central driver of PND pathogenesis [3, 4, 5]. Among the key mediators of neuroinflammatory processes, microglia—the resident immune cells of the central nervous system (CNS)—have garnered significant attention. These cells play a critical role in maintaining neuronal homeostasis, responding to injury, and regulating synaptic plasticity. However, aberrant microglial activation induces excessive production of pro‐inflammatory cytokines, oxidative stress, and synaptic dysfunction, all of which contribute to PND‐associated cognitive decline [6, 7, 8].

Modulating microglial activity represents a promising therapeutic approach for mitigating PND‐related cognitive deficits. Preclinical studies have demonstrated that targeting microglial activation attenuates neuroinflammatory responses and preserves cognitive function in animal models of PND [9, 10]. Advances in elucidating the molecular mechanisms governing microglial responses have identified potential therapeutic targets for pharmacological intervention.

This review provides a comprehensive analysis of microglial involvement in PND pathophysiology, detailing the mechanisms by which microglial activation contributes to cognitive dysfunction. It examines perioperative triggers, including surgical stress, anesthesia, and systemic inflammation, and evaluates current preclinical strategies aimed at modulating microglial activity for PND prevention and treatment. Additionally, it discusses the translational challenges associated with these therapeutic approaches, emphasizing the need for rigorous clinical validation. By delineating the intricate interplay between microglial activation and perioperative cognitive impairment, this discussion aims to inform the development of targeted interventions to mitigate PND and improve postoperative outcomes.

## 2 Overview of PND Pathophysiological Mechanisms

PND arise from a complex interplay of pathophysiological processes that collectively drive cognitive impairments in surgical patients. Elucidating these mechanisms is essential for developing effective preventive and therapeutic strategies. This section outlines the key pathophysiological contributors to PND.

### 2.1 Neuroinflammation

Neuroinflammation serves as a pivotal mechanism in PND pathogenesis. Surgical procedures trigger a systemic inflammatory response, characterized by elevated levels of pro‐inflammatory cytokines such as interleukin‐1β (IL‐1β), tumor necrosis factor‐alpha (TNF‐α), and interleukin‐6 (IL‐6). These cytokines can penetrate a compromised blood–brain barrier (BBB) or stimulate endothelial and peripheral immune cells to release additional inflammatory mediators within the CNS [11]. Microglia, the resident immune cells of the CNS, become activated in response, leading to the sustained production of pro‐inflammatory molecules that exacerbate neuronal dysfunction and apoptosis. Persistent or excessive neuroinflammation disrupts synaptic plasticity, a process fundamental to learning and memory, thereby contributing to PND‐associated cognitive decline [12].

### 2.2 BBB Disruption

The BBB plays a pivotal role in preserving CNS homeostasis, yet surgical trauma and anesthesia can compromise its integrity through mechanical stress, ischemia–reperfusion injury, and inflammatory mediator activity [13]. BBB disruption facilitates the infiltration of peripheral immune cells and the entry of neurotoxic substances into the brain parenchyma, further amplifying neuroinflammatory cascades. Additionally, loss of BBB integrity induces ionic imbalances and cerebral edema, exacerbating neuronal dysfunction and accelerating cognitive decline [14]. Restoring BBB integrity represents a promising therapeutic target for mitigating PND.

### 2.3 Neurotransmitter Imbalance

Neurotransmitter dysregulation is another critical contributor to PND. Cholinergic dysfunction, marked by reduced acetylcholine availability, impairs attention, memory, and executive function [15]. Similarly, excessive activation of N‐methyl‐D‐aspartate (NMDA) receptors within the glutamatergic system promotes excitotoxicity and neuronal injury [16]. Imbalances in monoaminergic neurotransmitters, including dopamine and serotonin, further contribute to mood disturbances and cognitive deficits in PND [17]. Pharmacological modulation of neurotransmitter systems may offer therapeutic potential in alleviating PND‐related cognitive impairments.

### 2.4 Oxidative Stress

Oxidative stress arises from an imbalance between reactive oxygen species (ROS) production and the brain's antioxidant defenses. Surgical interventions elevate ROS levels through ischemia–reperfusion injury, mitochondrial dysfunction, and inflammatory pathway activation [18]. Excessive ROS induce oxidative damage to proteins, lipids, and DNA, promoting neuronal apoptosis and synaptic dysfunction. Furthermore, oxidative stress exacerbates neuroinflammation by enhancing microglial activation and pro‐inflammatory cytokine release [19]. Antioxidant therapies hold promise in attenuating oxidative damage and preserving cognitive function in PND.

### 2.5 Mitochondrial Dysfunction

Mitochondria play a central role in cellular energy metabolism and apoptosis regulation. Surgical stress and anesthesia can impair mitochondrial function, leading to diminished ATP synthesis, excessive ROS production, and activation of apoptotic pathways [20]. Mitochondrial dysfunction disrupts neuronal metabolism and synaptic activity, exacerbating cognitive deficits. Moreover, dysregulated mitochondrial dynamics, including imbalances in fission and fusion processes, can induce neuronal structural abnormalities and compromise synaptic integrity [21]. Strategies aimed at preserving mitochondrial function hold promise for neuroprotection in the context of PND.

### 2.6 Synaptic Dysfunction and Neuroplasticity

Cognitive functions such as learning and memory rely on synaptic plasticity, including long‐term potentiation (LTP) and long‐term depression (LTD). PND is characterized by synaptic plasticity impairments driven by neuroinflammation, oxidative stress, and neurotransmitter imbalances. Pro‐inflammatory cytokines disrupt synaptic signaling by altering receptor trafficking and impairing synaptic transmission [22]. Additionally, oxidative damage to synaptic proteins and lipids compromises synaptic integrity and function [23]. Pharmacological and behavioral interventions that enhance synaptic plasticity may aid in restoring cognitive function in patients with PND.

### 2.7 Apoptosis and Neuronal Death

Apoptotic pathways contribute significantly to neuronal loss in PND. Surgical trauma and the subsequent inflammatory cascade activate both intrinsic and extrinsic apoptotic pathways [24]. The intrinsic pathway involves mitochondrial cytochrome c release and caspase activation, whereas the extrinsic pathway is mediated by death receptors such as Fas and TNF receptors [25]. Neuronal apoptosis in key cognitive regions, including the hippocampus and prefrontal cortex, accelerates cognitive decline [26]. Targeting apoptotic pathways through molecular interventions may help preserve neuronal populations and mitigate cognitive dysfunction in PND.

### 2.8 Genetic and Epigenetic Factors

Genetic predispositions and epigenetic modifications influence individual susceptibility to PND. Polymorphisms in genes encoding inflammatory cytokines, neurotransmitter receptors, and antioxidant enzymes modulate neuroinflammatory and oxidative responses to surgical stress. Epigenetic alterations, including DNA methylation, histone modifications, and microRNA regulation, can reshape gene expression in the CNS, affecting neuronal resilience and cognitive function [27]. Understanding these genetic and epigenetic determinants may facilitate the identification of high‐risk individuals and inform personalized therapeutic strategies.

### 2.9 Neuroendocrine Dysregulation

Surgical stress activates the hypothalamic–pituitary–adrenal (HPA) axis, elevating glucocorticoid levels such as cortisol. Chronic glucocorticoid exposure disrupts hippocampal function, suppressing neurogenesis and impairing synaptic plasticity, both of which are critical for cognition. Dysregulation of additional neuroendocrine pathways, including the sympathetic nervous system, further amplifies neuroinflammation and oxidative stress [28]. Modulating neuroendocrine responses to surgical stress may provide a viable approach to mitigating PND‐associated cognitive impairments.

### 2.10 Microbiota‐Gut‐Brain Axis

The microbiota‐gut‐brain axis constitutes a bidirectional communication network linking the gastrointestinal tract and the CNS through neural, endocrine, and immune pathways. Surgical interventions can disrupt gut microbiota composition, leading to dysbiosis and altered microbial metabolite production [29]. These microbial alterations influence neuroinflammation and compromise BBB integrity, ultimately affecting cognitive function [30]. Restoring microbial homeostasis through probiotics, prebiotics, or dietary interventions presents a potential strategy for mitigating PND by reducing neuroinflammatory responses and preserving cognitive integrity.

### 2.11 Sleep Disturbances

Postoperative sleep disturbances are prevalent and can significantly impede cognitive recovery. Sleep is essential for memory consolidation, synaptic homeostasis, and neuroplasticity [31]. Sleep fragmentation and diminished sleep quality exacerbate neuroinflammation, increase oxidative stress, and impair synaptic plasticity, collectively contributing to cognitive decline in PND [32]. Optimizing sleep quality in the perioperative period may enhance cognitive resilience and reduce the incidence of PND.

### 2.12 Vascular Contributions

Cerebral hypoperfusion and intraoperative ischemic events contribute to vascular‐mediated cognitive impairment, which shares pathological features with PND. Insufficient cerebral blood flow induces ischemic injury, BBB disruption, and subsequent neuroinflammation and oxidative stress [33]. Preexisting vascular risk factors, including hypertension, diabetes, and atherosclerosis, may exacerbate postoperative cognitive decline [34, 35, 36]. Ensuring adequate cerebral perfusion and managing vascular risk factors are critical for mitigating vascular contributions to PND.

Collectively, the pathophysiology of PND is multifaceted, involving neuroinflammation, BBB disruption, neurotransmitter imbalances, oxidative stress, mitochondrial dysfunction, synaptic and neuronal damage, genetic and epigenetic modifications, neuroendocrine dysregulation, microbiota–gut–brain axis alterations, sleep disturbances, and vascular contributions. These interconnected mechanisms underscore the complexity of PND and highlight the necessity for multifaceted preventive and therapeutic strategies.

## 3 Activation of Microglia Following PND

**FIGURE 1:**
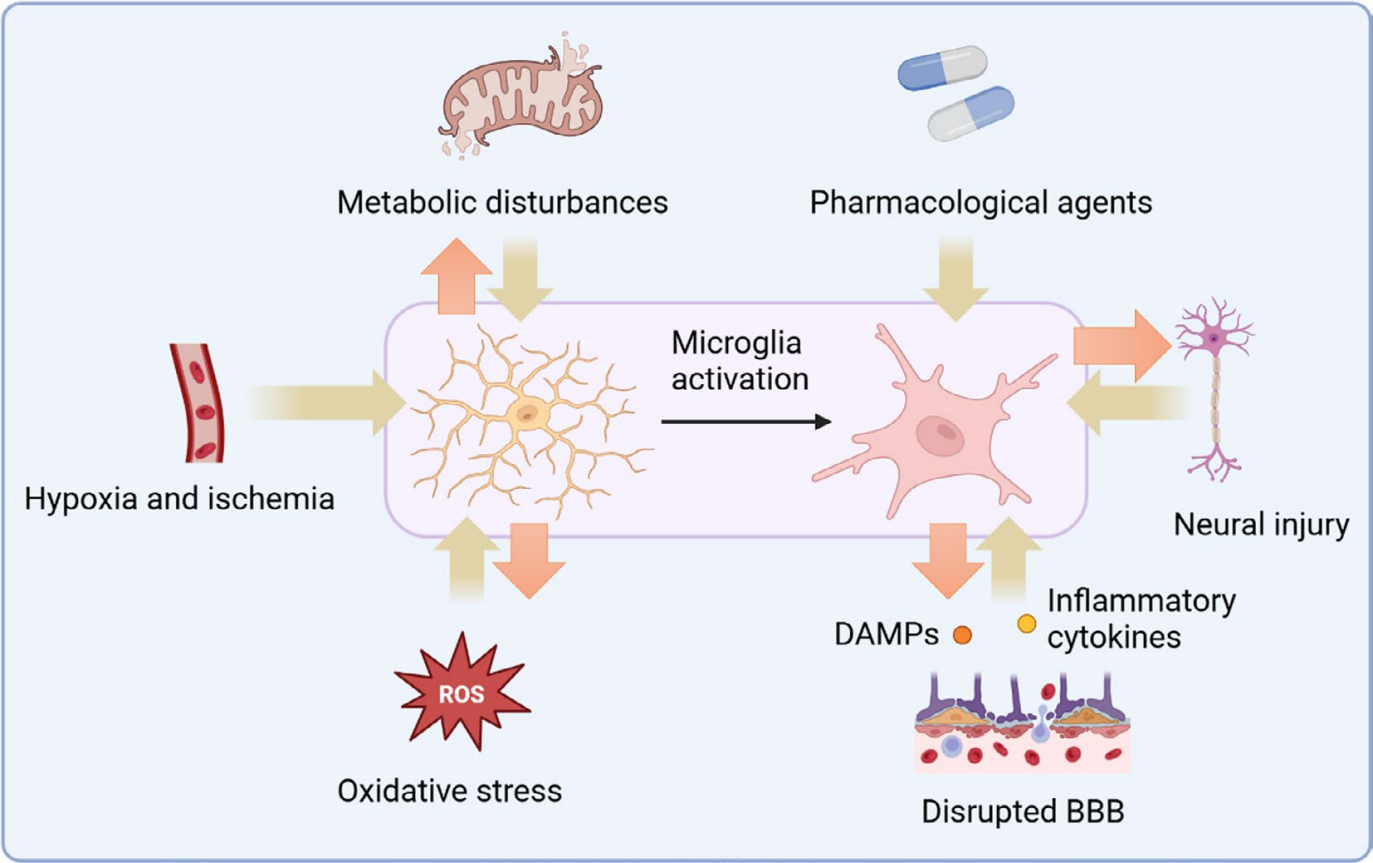
Mechanisms of microglial activation following PND. Following PND, microglial activation is driven by multiple converging factors. The inflammatory response induced by surgical trauma elevates systemic cytokines (e.g., IL‐1β, TNF‐α), which penetrate the CNS and activate microglia (See Section 3.1). Concurrently, DAMPs released from injured tissue engage microglial pattern recognition receptors, amplifying neuroinflammation (See Section 3.3). BBB disruption facilitates peripheral immune cell infiltration and neurotoxic substance entry, further stimulating microglia (See Section 3.2). Hypoxia and ischemia during surgery induce cellular stress and ROS production, promoting microglial activation (See Section 3.4). Neural injury releases intracellular components that exacerbate microglial responses (See Section 3.9), while metabolic disturbances alter microglial energy homeostasis, sustaining inflammation (See Section 3.10). Pharmacological agents, including anesthetics and opioids, modulate microglial activity, influencing neuroinflammatory outcomes (See Section 3.11). Together, these interconnected factors drive a pro‐inflammatory microglial phenotype that underlies the pathogenesis of PND.

Microglia, the primary immune sentinels of the CNS, are integral to maintaining neural homeostasis and responding to pathological stimuli. Perioperative events trigger a cascade of factors that converge to activate microglia, initiating neuroinflammatory responses that drive PND pathogenesis. This section delineates the principal triggers of microglial activation in PND (Figure 1).

### 3.1 Inflammatory Response

Surgical procedures invariably elicit a systemic inflammatory response, marked by elevated circulating levels of pro‐inflammatory cytokines such as IL‐1β, TNF‐α, and IL‐6 [37]. These cytokines reach the CNS via two primary mechanisms: direct penetration through a compromised BBB or indirect activation of endothelial and peripheral immune cells. Once within the CNS, they serve as potent stimuli for microglial activation [38]. In turn, activated microglia amplify neuroinflammation by secreting additional pro‐inflammatory mediators, establishing a self‐sustaining feedback loop that exacerbates neuronal dysfunction and cognitive impairment in PND [39].

### 3.2 BBB Disruption

BBB integrity is critical for shielding the CNS from peripheral inflammatory insults. However, surgical trauma and physiological stress compromise BBB function through mechanical disruption, ischemia–reperfusion injury, and the actions of inflammatory mediators [40]. BBB breakdown permits the infiltration of peripheral immune cells, such as monocytes and neutrophils, and the entry of neurotoxic substances into the brain parenchyma [41]. These infiltrating elements act as additional microglial activators, intensifying neuroinflammation. Moreover, compromised BBB integrity disrupts ionic homeostasis and induces cerebral edema, exacerbating neuronal dysfunction and sustaining microglial activation.

### 3.3 Tissue Damage and Release of DAMPs

Tissue injury resulting from surgical interventions leads to the release of damage‐associated molecular patterns (DAMPs), including high‐mobility group box 1 (HMGB1), heat shock proteins, and extracellular ATP [42]. These endogenous danger signals engage pattern recognition receptors (PRRs) on microglia, such as Toll‐like receptors (TLRs) and NOD‐like receptors (NLRs), triggering intracellular signaling cascades that activate microglia and promote the secretion of pro‐inflammatory cytokines and ROS [43]. This process not only amplifies neuroinflammation but also contributes to neuronal apoptosis and synaptic dysfunction, hallmark features of PND.

### 3.4 Hypoxia and Ischemia

Surgical procedures, particularly those involving significant blood loss or prolonged anesthesia, can induce transient cerebral hypoxia and ischemia, further exacerbating microglial activation. Hypoxia‐driven cellular stress and energy deficits activate microglia via hypoxia‐inducible factors (HIFs) and other stress‐responsive pathways [44]. Ischemia–reperfusion injury, a frequent consequence of perioperative hypoxia, leads to excessive ROS generation and the release of DAMPs, further stimulating microglial activation [45]. In this state, microglia contribute to neuronal damage by producing inflammatory mediators and phagocytosing stressed but viable neurons, thereby worsening cognitive deficits associated with PND.

### 3.5 Stress Responses

The perioperative period imposes substantial physiological and psychological stress, activating the hypothalamic–pituitary–adrenal (HPA) axis and the sympathetic nervous system [28, 46]. Elevated glucocorticoid levels, such as cortisol, and catecholamines, including norepinephrine, modulate microglial function, with chronic glucocorticoid exposure priming microglia to exhibit heightened sensitivity to subsequent inflammatory stimuli. Additionally, catecholamines influence microglial activation states, promoting a pro‐inflammatory phenotype [47]. These stress‐induced alterations contribute to the persistent neuroinflammatory milieu characteristic of PND.

### 3.6 Release of Bioactive Molecules

Postoperative physiological disturbances lead to the release of bioactive molecules, including neurotransmitters, cytokines, and lipid mediators, which further influence microglial activation. Elevated glutamate levels resulting from excitotoxicity activate microglial receptors, such as NMDA receptors, driving inflammatory responses [16]. Similarly, increased extracellular ATP disrupts purinergic signaling, engaging P2X7 receptors on microglia and triggering NLRP3 inflammasome assembly, leading to IL‐1β release [48]. These bioactive mediators establish a direct link between neuronal distress and microglial activation, perpetuating neuroinflammation in PND.

### 3.7 Peripheral Nerve Stimulation

Peripheral nerve injury or stimulation during surgical trauma generates distress signals that propagate to the CNS through neuropeptides and other signaling molecules. These signals activate afferent neurons, stimulating pro‐inflammatory cytokine release and immune cell recruitment into the CNS [49]. In response, microglia transition to an activated state, adopting a pro‐inflammatory phenotype that exacerbates neuroinflammation in PND. Additionally, peripheral nerve stimulation enhances sympathetic outflow, further influencing microglial activation through catecholamine‐mediated pathways [50].

### 3.8 Infection and Sepsis

Postoperative infections, particularly sepsis, represent potent systemic inflammatory triggers that profoundly impact the CNS. Circulating pathogens and their molecular patterns, such as lipopolysaccharides (LPS) from Gram‐negative bacteria, can infiltrate the CNS, especially in the presence of BBB compromise. These pathogen‐associated molecular patterns (PAMPs) engage microglial PRRs, initiating robust microglial activation and an amplified inflammatory response [51]. Sepsis‐induced neuroinflammation is marked by excessive cytokine release, ROS production, and mitochondrial dysfunction within microglia, collectively driving neuronal injury and cognitive deficits characteristic of PND.

### 3.9 Neural Injury and Axonal Damage

Surgical procedures can inadvertently induce direct neural injury or axonal damage, releasing intracellular components that function as DAMPs. These molecules activate microglia through PRRs, mirroring inflammatory responses observed in other forms of tissue damage. Axonal disruption further compromises neural circuitry and synaptic integrity, prompting compensatory microglial responses aimed at debris clearance and repair. However, excessive or prolonged microglial activation fosters a persistent pro‐inflammatory state, leading to aberrant synaptic pruning and neuronal loss, thereby exacerbating cognitive deficits in PND.

### 3.10 Metabolic and Epigenetic Regulation

Surgical interventions also disrupt metabolic homeostasis, altering glucose metabolism, insulin signaling, and electrolyte balance within the CNS. Hypoglycemia, hyperglycemia, and ionic imbalances, particularly in calcium and potassium, induce cellular stress and trigger microglial activation [52]. Metabolic disturbances exacerbate oxidative stress and mitochondrial dysfunction, promoting a sustained pro‐inflammatory microglial phenotype. A study employing single‐cell RNA sequencing revealed widespread expression changes of metabolism‐related genes in hippocampal microglial cells during PND, with these metabolic alterations correlating with pro‐inflammatory activation [53]. Metabolic changes could extensively regulate the expression of inflammation‐related genes through histone modifications [54, 55], potentially contributing to the intensification of microglial inflammation during PND.

### 3.11 Pharmacological Agents

Anesthetic and analgesic agents exert direct and indirect effects on microglial activation [56]. Certain anesthetics, such as isoflurane and sevoflurane, modulate microglial activity, either promoting anti‐inflammatory responses or exacerbating neuroinflammation depending on dosage and exposure context [57]. Opioids, frequently administered for perioperative pain management, engage opioid receptors on microglia, potentially sustaining a chronic inflammatory state [58]. Understanding the pharmacological impact on microglial dynamics is essential for refining perioperative care strategies to mitigate PND risk.

### 3.12 Age‐Related Factors

The elderly population, inherently more susceptible to PND, exhibits age‐related alterations in microglial function. Aging microglia exist in a primed state, characterized by heightened reactivity to inflammatory stimuli [59]. This exaggerated responsiveness amplifies microglial activation following surgical stress, intensifying neuroinflammatory cascades. Additionally, age‐associated declines in BBB integrity and increased oxidative stress further predispose older individuals to prolonged microglial activation and exacerbated postoperative cognitive impairments [60].

### 3.13 Genetic Susceptibility

Genetic predisposition plays a pivotal role in determining an individual's susceptibility to microglial activation. Polymorphisms in genes encoding inflammatory cytokines, PRRs, and signaling molecules modulate microglial responsiveness to perioperative stimuli. For instance, variations in the TLR4 gene influence microglial activation in response to endotoxins, affecting the severity of neuroinflammation and the likelihood of developing PND [61]. Identifying such genetic susceptibilities may facilitate personalized therapeutic strategies aimed at attenuating microglial activation and mitigating PND risk.

In summary, microglial activation in the perioperative setting arises from a complex interplay of systemic inflammation, BBB disruption, tissue injury, hypoxia, stress responses, bioactive molecule release, peripheral nerve stimulation, infections, neural damage, metabolic disturbances, pharmacological exposures, aging‐related changes, and genetic factors. These converging triggers induce a pro‐inflammatory microglial phenotype that sustains neuroinflammation, impairs synaptic function, and drives neuronal injury, ultimately contributing to the cognitive deficits characteristic of PND [62]. A comprehensive understanding of these activation mechanisms is fundamental for designing targeted interventions that modulate microglial responses and mitigate neurocognitive complications following surgery.

## 4 Role of Microglial Activation in the Pathophysiological Mechanisms of PND

**FIGURE 2:**
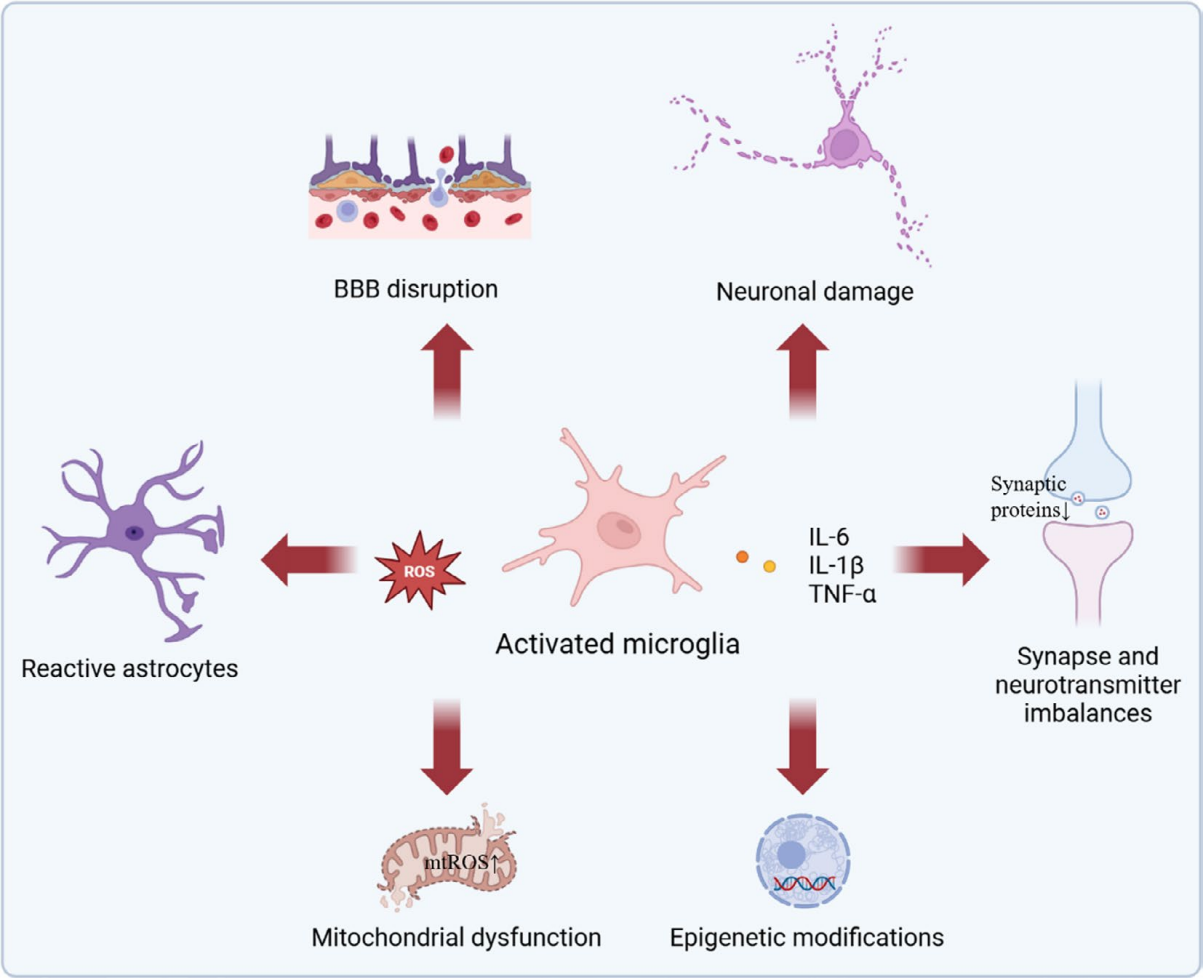
Role of microglial activation in the pathophysiological mechanisms of PND. Microglial activation plays a pivotal role in the progression of PND through multiple interconnected mechanisms. Upon activation, microglia release pro‐inflammatory cytokines (e.g., IL‐1β, IL‐6, TNF‐α), initiating and sustaining neuroinflammation (See Section 4.1). This inflammatory milieu disrupts synaptic function and neurotransmitter balance, impairing neural connectivity and cognitive processes (See Section 4.3 and 4.5). Concurrently, activated microglia generate reactive oxygen and nitrogen species, inducing oxidative stress that damages neuronal components (See Section 4.2). They also promote phenotypic transformation of astrocytes into reactive states, amplifying neuroinflammation (See Section 4.7). Microglial‐derived factors compromise BBB integrity, facilitating peripheral immune infiltration and further inflammation (See Section 4.6). Additionally, microglial activation impairs mitochondrial function in neurons, leading to energy deficits and apoptotic signaling (See Section 4.8). Epigenetic modifications triggered by microglial signals reprogram gene expression, perpetuating pathological changes (See Section 4.9). These processes result in neuronal damage, driving the cognitive decline characteristic of PND (See Section 4.4). This integrated network underscores microglia as central mediators in PND pathophysiology and highlights potential therapeutic targets.

PND encompass a spectrum of cognitive impairments arising in the context of surgical procedures and anesthesia, ranging from subtle cognitive dysfunction to more severe manifestations such as postoperative delirium and persistent cognitive decline. Accumulating evidence underscores the central role of microglial activation in the pathophysiology of PND [63]. As the resident immune cells of the CNS, microglia serve as primary mediators of neuroinflammation and are implicated in various neurodegenerative and neuroinflammatory conditions. This section delineates the multifaceted contributions of activated microglia to PND pathology, highlighting key mechanisms such as inflammatory cytokine release, oxidative stress, synaptic dysfunction, neuronal damage, neurotransmitter imbalances, and BBB disruption [64] (Figure 2).

### 4.1 Release of Inflammatory Cytokines

Microglial activation is a hallmark of neuroinflammation, characterized by the secretion of pro‐inflammatory cytokines such as IL‐1β, IL‐6, and TNF‐α, which orchestrate inflammatory responses within the CNS [65]. In PND, surgical trauma and anesthesia‐induced stressors precipitate microglial activation, leading to elevated cytokine levels. IL‐1β has been implicated in impaired synaptic plasticity and cognitive dysfunction, while IL‐6 and TNF‐α contribute to neuronal apoptosis and synaptic impairment [39]. The sustained release of these cytokines fosters a chronic inflammatory state, perpetuating postoperative cognitive decline. Furthermore, cytokine‐mediated modulation of neuronal excitability and neurotransmitter system disruptions exacerbate cognitive impairments.

### 4.2 Oxidative Stress

Activated microglia are major sources of ROS and reactive nitrogen species (RNS), contributing to oxidative stress within the CNS. Oxidative stress arises from an imbalance between ROS/RNS production and antioxidant defense mechanisms [66]. In PND, heightened oxidative stress promotes lipid peroxidation, protein oxidation, and DNA damage, collectively impairing neuronal function and viability [67]. Moreover, ROS and RNS activate additional inflammatory pathways, creating a self‐perpetuating cycle of microglial activation and neuroinflammation. This oxidative environment not only directly damages neurons but also disrupts synaptic integrity and plasticity, further exacerbating cognitive deficits [68].

### 4.3 Synaptic Function Alterations

Microglial activation profoundly impacts synaptic structure and function, which are fundamental to learning and memory. Activated microglia secrete factors such as brain‐derived neurotrophic factor (BDNF) and complement system components that influence synaptic plasticity [38]. Dysregulation of these factors leads to excessive synaptic pruning, particularly in cognition‐critical regions such as the hippocampus and prefrontal cortex [69]. Additionally, inflammatory cytokines alter neurotransmitter receptor function and synaptic signaling, further impairing neural connectivity. These synaptic disruptions compromise neuronal circuit function, contributing to the cognitive deficits characteristic of PND.

### 4.4 Neuronal Damage

The interplay between microglia and neurons is a key determinant of neuronal survival and functional integrity. Pro‐inflammatory cytokines, ROS/RNS, and excitotoxic neurotransmitters released by activated microglia induce neuronal apoptosis and necrosis [12]. Excessive glutamate release and impaired uptake result in excitotoxicity, leading to calcium overload and apoptotic pathway activation. Additionally, TNF‐α released by microglia can trigger neuronal apoptosis through receptor‐mediated signaling cascades [70]. The ensuing neuronal damage not only diminishes cognitive capacity but also disrupts neural networks essential for higher‐order cognitive functions. Chronic microglial activation and subsequent neuronal loss contribute to the persistent cognitive impairments observed in PND [71].

### 4.5 Neurotransmitter Imbalances

Microglial activation disrupts neurotransmitter homeostasis, a critical determinant of cognitive function. Activated microglia modulate key neurotransmitters, including glutamate, γ‐aminobutyric acid (GABA), dopamine, and acetylcholine. By producing and releasing glutamate, microglia contribute to excitotoxicity and synaptic dysfunction, exacerbating neuronal stress. Additionally, they influence GABAergic transmission by altering receptor expression and modulating GABA synthesis, thereby affecting inhibitory signaling [72]. Dopaminergic and cholinergic systems, essential for cognitive processes, are similarly vulnerable to disruption by microglial‐derived inflammatory mediators. These neurotransmitter imbalances impair synaptic transmission and neural network dynamics, ultimately compromising attention, memory, and executive function [73].

### 4.6 BBB Disruption

BBB integrity is fundamental to CNS homeostasis, but microglial activation contributes to its disruption through the secretion of matrix metalloproteinases (MMPs), cytokines, and other inflammatory mediators. MMPs degrade tight junction proteins, impairing BBB selectivity and increasing permeability [74]. This disruption permits peripheral immune cell infiltration and facilitates neuroinflammatory amplification. In PND, surgical stress and subsequent microglial activation sustain a pathological cycle of BBB compromise and neuroinflammation, allowing neurotoxic substances and immune cells to penetrate the CNS, exacerbating neuronal damage and cognitive decline [75].

### 4.7 Interaction With Other Glial Cells

Microglia interact dynamically with other glial cells, including astrocytes and oligodendrocytes, shaping the neuroinflammatory landscape. Activated microglia induce a reactive astrocyte phenotype, further amplifying neuroinflammation and synaptic dysfunction [76]. This interplay exacerbates the inflammatory milieu, perpetuating sustained neuroinflammation and cognitive impairment. Additionally, microglial activation affects oligodendrocyte function and myelination, potentially disrupting neural transmission and impairing cognitive processing.

### 4.8 Mitochondrial Dysfunction

Emerging evidence links microglial activation to mitochondrial dysfunction in neurons. Microglia‐derived factors impair mitochondrial dynamics, bioenergetics, and mitophagy, leading to compromised ATP production, excessive ROS generation, and activation of apoptotic pathways [77]. In PND, these cumulative mitochondrial deficits exacerbate neuronal dysfunction, contributing to progressive cognitive impairment.

### 4.9 Epigenetic Modifications

Microglial activation induces epigenetic modifications that reprogram gene expression in neurons and other glial cells. These modifications, including DNA methylation, histone acetylation/deacetylation, and non‐coding RNA regulation, lead to sustained changes in the expression of genes governing synaptic plasticity, neuronal survival, and inflammatory responses. Such epigenetic reprogramming may underlie the persistent cognitive impairments observed in PND, emphasizing the role of microglial activation in shaping the CNS epigenetic landscape during the perioperative period [78].

Collectively, microglial activation is a central driver of PND pathophysiology. Through the release of pro‐inflammatory cytokines, oxidative stress generation, synaptic dysfunction, neuronal injury, neurotransmitter imbalances, and BBB disruption, activated microglia orchestrate a cascade of pathological processes leading to cognitive impairment [79]. Additionally, microglial interactions with astrocytes and oligodendrocytes, contributions to mitochondrial dysfunction, and induction of epigenetic modifications further exacerbate neuroinflammation and cognitive decline in PND. Targeting microglial activation and its downstream effects represents a compelling therapeutic strategy for mitigating the neurocognitive sequelae of surgical interventions [80].

## 5 Preclinical Studies on Targeting Microglia for the Treatment of PND

PND pose a significant clinical challenge, particularly among elderly surgical patients, due to their prolonged cognitive consequences and association with increased morbidity. Emerging preclinical studies have identified microglial modulation as a promising intervention for PND prevention and treatment. This section reviews recent advancements in targeting microglia to attenuate PND, encompassing strategies such as microglial depletion, polarization modulation, and inhibition of specific inflammatory pathways.

### 5.1 Microglial Depletion

One of the primary strategies explored in preclinical models involves microglial depletion to evaluate their role in PND pathogenesis [81]. Xu et al. employed a sevoflurane‐induced neurotoxicity (SIN) rat model, demonstrating that prolonged anesthesia activates the NF‐κB pathway, driving neuroinflammation and cognitive dysfunction. Microglial depletion significantly attenuated neuroinflammation and complement activation, preventing synaptic loss and rescuing cognitive deficits [38]. Similarly, Feng et al. utilized colony‐stimulating factor 1 receptor (CSF1R) inhibitors to deplete microglia in adult mice subjected to surgical trauma, observing robust protection against PND, including preserved cognitive function and reduced hippocampal inflammatory mediator levels, even in diet‐induced obese mice—a condition known to exacerbate PND [82]. These findings reinforce the pivotal role of microglia in PND and validate microglial depletion as a potential therapeutic intervention.

### 5.2 Modulation of Microglial Polarization

Shifting microglial polarization from a pro‐inflammatory (M1) to an anti‐inflammatory (M2) phenotype has emerged as a promising approach to mitigating PND‐associated neuroinflammation.

#### 5.2.1 Metabolic Reprogramming

Luo et al. investigated metabolic reprogramming in microglial polarization within a PND model induced by surgical trauma in aged mice. Their study revealed that surgical injury drives microglial metabolism from oxidative phosphorylation to glycolysis, promoting an M1 pro‐inflammatory phenotype. Administration of the glycolytic inhibitor 2‐deoxy‐D‐glucose (2‐DG) reversed this metabolic shift, suppressing M1 polarization and reducing pro‐inflammatory cytokine production. Notably, 2‐DG treatment ameliorated hippocampus‐dependent cognitive deficits, highlighting metabolic modulation as a viable therapeutic avenue [37].

#### 5.2.2 Pharmacological Agents

Jiang et al. explored the neuroprotective potential of lipoxin A4 (LXA4), a lipid‐derived anti‐inflammatory mediator. In a mouse model of PND, LXA4 pre‐administration significantly attenuated surgery‐induced cognitive impairment, neuroinflammation, and microglial activation [83]. In vitro experiments using BV2 microglial cells confirmed that LXA4 inhibited M1 polarization while promoting the M2 anti‐inflammatory phenotype via activation of the SIRT1/NF‐κB pathway [65].

Another promising pharmacological agent, galectin‐1, was evaluated by Shen et al. in aged mice undergoing laparotomy under isoflurane anesthesia. Galectin‐1 administration effectively reduced cognitive dysfunction and suppressed microglial activation by downregulating IRAK1 expression, thereby diminishing neuroinflammation [84]. These findings collectively underscore the therapeutic potential of pharmacological modulators in directing microglial polarization toward a neuroprotective state, offering a promising strategy for mitigating PND.

#### 5.2.3 Receptor‐Mediated Modulation

Chen et al. investigated the role of histamine H2/H3 receptors in modulating microglia‐driven inflammation in PND. Their in vivo studies demonstrated that receptor activation suppressed microglial activation, attenuated pro‐inflammatory cytokine release, and promoted M2 polarization through the PI3K/AKT/FoxO1 pathway [62]. Similarly, Tang et al. utilized AAV‐mediated knockdown of triggering receptor expressed on myeloid cells‐1 (TREM1) in hippocampal microglia, which mitigated M1 polarization, reduced pro‐inflammatory cytokine levels, and improved cognitive outcomes in sevoflurane‐induced PND models [85].

Li et al. further highlighted the immunoregulatory function of IL‐33 in PND, demonstrating that its administration shifted microglial polarization from M1 to M2. IL‐33 treatment not only alleviated neuroinflammation but also preserved synaptic plasticity and cognitive function, underscoring cytokine‐mediated microglial modulation as a promising therapeutic strategy [86].

### 5.3 Inhibition of Inflammatory Pathways in Microglia

Preclinical research has also emphasized targeting specific inflammatory signaling pathways within microglia for PND intervention.

#### 5.3.1 NF‐κB and NLRP3 Pathways

Xu et al. delineated the involvement of NF‐κB signaling in sevoflurane‐induced neuroinflammation and cognitive impairment, showing that prolonged anesthesia activated NF‐κB, driving inflammatory cytokine upregulation and exacerbating microglial activation. Suppressing this pathway via microglial depletion or complement component C1q neutralization effectively mitigated neuroinflammation and cognitive deficits, establishing NF‐κB as a key contributor to PND pathogenesis [38].

Similarly, Zhang et al. investigated the NLRP3 inflammasome, a central regulator of microglial inflammatory responses, and identified dexmedetomidine (DEX), an α2‐adrenergic receptor agonist, as an inhibitor of NLRP3 activation via the autophagy‐ubiquitin pathway. DEX treatment reduced mature IL‐1β secretion and dampened inflammatory signaling, thereby ameliorating cognitive impairments in PND models [87]. These findings reinforce the therapeutic potential of targeting microglial inflammatory pathways to counteract PND‐associated neuroinflammation.

#### 5.3.2 Complement System Modulation

The complement system, particularly the C3/C3a receptor signaling axis, has also been implicated in synaptic elimination and cognitive dysfunction in PND. Xiong et al. reported that orthopedic surgery elevated C3 levels and C3a receptor expression in hippocampal astrocytes and microglia. Pharmacological blockade of the C3a receptor attenuated neuroinflammation, preserved synaptic integrity, and improved hippocampal‐dependent memory, highlighting the complement system's role in PND pathology and the therapeutic efficacy of its inhibition [88].

### 5.4 Pharmacological Agents Targeting Microglia

Beyond specific signaling pathways, various pharmacological agents have been identified for their ability to modulate microglial activity and alleviate PND.

#### 5.4.1 Gastrodin and Liproxstatin‐1

Wang et al. investigated the neuroprotective effects of gastrodin (GAS), a natural plant‐derived compound, in a PND mouse model. GAS treatment suppressed microglial activation, attenuated pro‐inflammatory cytokine production, and inhibited GSK‐3β and Tau phosphorylation. These molecular effects preserved synaptic integrity and improved cognitive performance, underscoring GAS as a potential therapeutic agent for PND [89]. Similarly, Li et al. explored ferroptosis, an iron‐dependent form of cell death, in PND pathogenesis. Administration of the ferroptosis inhibitor liproxstatin‐1 ameliorated memory deficits, reduced microglial activation, and mitigated oxidative stress, highlighting ferroptosis inhibition as a neuroprotective strategy in PND [66].

#### 5.4.2 *Sigesbeckia orientalis* and Other Natural Compounds

Natural compounds have also garnered attention for their anti‐inflammatory properties [90]. Chu et al. isolated an active fraction from *Sigesbeckia orientalis* L. (SO), demonstrating its potent anti‐neuroinflammatory effects in both microglial cultures and PND mouse models. Treatment with the SO‐derived fraction suppressed microglial activation, decreased pro‐inflammatory cytokine levels, and preserved dendritic spine density, leading to improved cognitive function without adverse bleeding effects. The presence of bioactive flavonoids and terpenoids further underscores the therapeutic potential of natural compounds in PND intervention [91].

### 5.5 Genetic and Molecular Approaches

Genetic modulation of microglial‐related genes provides another avenue for PND treatment.

#### 5.5.1 PTEN Knockdown

Shen et al. investigated the role of phosphatase and tensin homolog deleted on chromosome ten (PTEN) in microglial activation and cognitive dysfunction in PND. Hippocampal PTEN knockdown attenuated microglial activation, reduced pro‐inflammatory cytokine expression, and mitigated cognitive deficits following surgery, positioning PTEN as a key regulator of microglia‐driven neuroinflammation and a potential genetic target for PND therapy [92].

#### 5.5.2 CD200‐CD200R1 Signaling

Ma et al. examined the CD200‐CD200R1 signaling axis, a key modulator of microglial activation. Activation of CD200R1 via CD200‐Fc injection in a PND mouse model suppressed neuroinflammation and cognitive decline by enhancing PSD‐95 expression and downregulating pro‐inflammatory cytokines, further demonstrating the therapeutic viability of targeting this pathway to regulate microglial activity [93].

### 5.6 Microbiome‐Related Interventions

Emerging evidence highlights a critical interplay between gut microbiota and microglial function in PND [94]. Wen et al. demonstrated that acetate, a short‐chain fatty acid produced by gut microbiota, inhibited microglial activation via the GPR43 receptor, thereby dampening neuroinflammation and preventing memory deficits in PND models. Moreover, modulation of the gut microbiome through fecal microbiota transplantation or probiotic supplementation altered microglial activity and conferred neuroprotection, suggesting the microbiome‐gut‐brain axis as a novel therapeutic target for PND [95].

### 5.7 Other Therapeutic Targets and Strategies

#### 5.7.1 BBB Integrity

Inhibition of matrix metalloproteinases (MMP‐2 and MMP‐9) has been shown to preserve BBB integrity and attenuate glial activation in PND models. Ji et al. demonstrated that pharmacological suppression of MMP‐2 and MMP‐9 alleviated anesthesia‐ and surgery‐induced cognitive deficits by maintaining tight junction protein expression and reducing microglial and astrocyte activation [96].

#### 5.7.2 Toll‐Like Receptors (TLRs)

Wei et al. investigated the TLR4/MyD88/NF‐κB signaling cascade in microglial‐mediated neuroinflammation and cognitive dysfunction. Their findings indicate that free heme, released during RBC transfusion, activates this pathway in microglia, triggering neuroinflammation and neuronal apoptosis. Inhibition of TLR4 signaling using hemopexin or pathway‐specific inhibitors effectively mitigated cognitive deficits, highlighting TLR4‐mediated neuroinflammation as a potential therapeutic target in PND [97]. Preclinical studies underscore microglial modulation as a promising therapeutic strategy for PND (Table 1). Approaches such as microglial depletion, M1‐to‐M2 polarization, suppression of inflammatory pathways (e.g., NF‐κB, NLRP3), and complement system modulation have demonstrated efficacy in ameliorating neuroinflammation and cognitive impairment in animal models. Pharmacological agents and genetic interventions, including PTEN suppression and CD200‐CD200R1 activation, further reinforce the pivotal role of microglia in PND pathogenesis [98]. Additionally, the gut microbiome's influence on microglial function presents an emerging therapeutic avenue. These findings suggest clinical applicability, with future research focusing on validation and the development of targeted therapies.

**TABLE 1:** Strategies of targeting microglia for the treatment of PND.

Therapeutic target	Animal model type	Related research finding	References
Microglial depletion	Sevoflurane‐induced rat PND model	Depleting microglia led to reduced neuroinflammation, complement activation, and synaptic loss, ultimately improving cognitive function	Xu et al. [38]
Microglial depletion	Anesthesia and surgery‐induced mouse PND model	Microglia depletion via CSF1R inhibitors in adult mice undergoing surgical trauma prevented cognitive decline and reduced hippocampal inflammation, even in obese mice	Feng et al. [82]
Modulation of microglial polarization	Anesthesia and surgery‐induced mouse PND model	After surgical trauma in aged mice, microglia shifted to glycolysis, promoting a pro‐inflammatory M1 phenotype; inhibiting glycolysis reversed this shift, reducing inflammation, and improving cognitive function	Luo et al. [37]
Modulation of microglial polarization	Anesthesia and surgery‐induced mouse PND model	Pre‐treatment with LXA4 reduced neuroinflammation, microglial activation, and cognitive deficits, promoting the anti‐inflammatory M2 phenotype	Jiang et al. [65]
Modulation of microglial polarization	Anesthesia and surgery‐induced mouse PND model	Galectin‐1 treatment in aged mice after laparotomy improved cognitive function and reduced microglial activation by inhibiting IRAK1 expression	Shen et al. [84]
Modulation of microglial polarization	Anesthesia and surgery‐induced rat PND model	Activation of histamine 2/3 receptors reduced microglial activation and cytokine production, shifting microglia toward a protective M2 phenotype, thereby improving cognitive function	Chen et al. [62]
Modulation of microglial polarization	Sevoflurane‐induced mouse PND model	Inhibiting TREM1 in hippocampal microglia decreased M1 polarization and pro‐inflammatory cytokines, leading to improved cognitive outcomes	Tang et al. [85]
Modulation of microglial polarization	Anesthesia and surgery‐induced mouse PND model	IL‐33 treatment shifted microglial polarization from M1 to M2, alleviating neuroinflammation and preserving cognitive function	Li et al. [86]
Inhibition of inflammatory pathways in microglia	Sevoflurane‐induced rat PND model	Prolonged anesthesia activated the NF‐κB pathway, driving neuroinflammation and cognitive deficits; inhibiting this pathway reduced inflammation and improved outcomes	Xu et al. [38]
Inhibition of inflammatory pathways in microglia	Anesthesia and surgery‐induced mouse PND model	Dexmedetomidine inhibited the NLRP3 inflammasome via autophagy, reducing IL‐1β production and alleviating cognitive deficits	Zhang et al. [87]
Inhibition of inflammatory pathways in microglia	Anesthesia and surgery‐induced mouse PND model	C3a receptor blockade after orthopedic surgery preserved hippocampal function and alleviated cognitive impairment by reducing neuroinflammation and maintaining synaptic integrity	Xiong et al. [88]
Inhibition of inflammatory pathways in microglia	Free heme‐induced rat PND model	Inhibiting TLR4/MyD88/NF‐κB signaling in microglia with hemopexin or specific inhibitors reduced cognitive deficits	Wei et al. [97]
Pharmacological agents targeting microglia	Anesthesia and surgery‐induced mouse PND model	GAS suppressed microglial activation, reduced inflammation, improved synaptic function, and alleviated cognitive dysfunction	Wang et al. [89]
Pharmacological agents targeting microglia	LPS‐induced mouse PND model	Inhibition of ferroptosis with liproxstatin‐1 alleviated memory deficits and reduced microglial activation, playing a neuroprotective role	Li et al. [66]
Pharmacological agents targeting microglia	Anesthesia and surgery‐induced mouse PND model	An active fraction from *Sigesbeckia orientalis* L. reduced microglial activation, lowered inflammation, and improved cognitive outcomes	Chu et al. [91]
Genetic and molecular approaches	Anesthesia and surgery‐induced mouse PND model	Knockdown of PTEN in the hippocampus reduced microglial activation and inflammation, leading to improved cognitive function	Shen et al. [92]
Genetic and molecular approaches	Anesthesia and surgery‐induced mouse PND model	Activation of the CD200‐CD200R1 pathway reduced neuroinflammation and cognitive decline by promoting synaptic function and reducing inflammation	Ma et al. [93]
Microbiome‐related interventions	Anesthesia and surgery‐induced mouse PND model	Acetate, produced by gut microbiota, suppressed microglial activation and neuroinflammation, with potential therapeutic effects on cognitive decline	Wen et al. [95]
BBB integrity maintain	Anesthesia and surgery‐induced mouse PND model	Inhibition of MMP‐2 and MMP‐9 protected against cognitive deficits by maintaining blood–brain barrier integrity and reducing glial activation after surgery	Ji et al. [96]

## 6 Clinical Translation of Targeting Microglia in the Treatment of PND

Translating preclinical insights into clinical applications targeting microglia for PND treatment represents an innovative approach with the potential to revolutionize perioperative care. This section explores the feasibility of clinical translation, associated challenges, and future directions.

### 6.1 Potential for Clinical Translation

Microglia's critical role in PND pathophysiology positions them as prime therapeutic targets. Agents such as minocycline, known for their microglial‐modulating properties, have demonstrated efficacy in reducing neuroinflammation and cognitive decline in neurological disorders and may be repurposed for PND treatment. Advances in biotechnology have facilitated the development of novel therapeutics, including monoclonal antibodies targeting microglial receptors or signaling pathways, such as CSF1R inhibitors capable of depleting or modulating microglial activity. The clinical potential of these therapies depends on their ability to suppress maladaptive microglial responses while preserving essential homeostatic functions within the CNS. Precision modulation of microglial activation states or selective inhibition of inflammatory pathways, such as the NLRP3 inflammasome, could offer targeted strategies to mitigate PND pathology while preserving cognitive function [99].

In recent clinical trials, significant advancements have been made with investigational drugs targeting CSF1R and NLRP3 pathways, enhancing their translational relevance in oncology and critical care. For instance, PLX3397, a CSF1R inhibitor, has demonstrated promising results in treating tenosynovial giant cell tumors (TGCT), showing a 39% overall response rate in patients who were not candidates for surgical intervention [100]. Additionally, NLRP3 inhibitors, such as MCC950, have shown potential in mitigating ventilator‐induced lung injury by suppressing NLRP3 inflammasome activation, thereby reducing pulmonary inflammation [101]. Although clinical trials involving PLX3397 and MCC950 in the context of PND have yet to be initiated, these findings underscore the importance of targeting these pathways to improve patient outcomes across various clinical settings, paving the way for future clinical research directions and therapeutic strategies in PND.

### 6.2 Challenges in Clinical Translation

While targeting microglia presents a compelling theoretical framework for PND treatment, several critical challenges must be addressed to facilitate clinical translation. A primary hurdle lies in the multifaceted nature of PND pathophysiology, which extends beyond microglial activation to encompass intricate crosstalk between microglia, neurons, and astrocytes. Additionally, systemic stress responses triggered by surgical interventions introduce further complexity, necessitating a more comprehensive therapeutic approach rather than a singular target strategy.

Another major obstacle is the restrictive permeability of the BBB, which significantly impedes the effective delivery of microglia‐targeting agents. Many promising therapeutics struggle to penetrate the CNS, necessitating the development of advanced drug delivery systems or molecular modifications to enhance BBB transit. Nanoparticle‐based delivery platforms offer a viable solution, leveraging optimized particle size and surface modifications to facilitate BBB passage and achieve precise microglial targeting [102]. For instance, nanoparticles functionalized with transferrin receptor‐targeting antibodies have demonstrated enhanced permeability across the BBB, significantly improving CNS delivery [103]. Engineered nanoparticles evade immune clearance, efficiently traverse the BBB, and directly release therapeutic agents within microglia [104]. By efficiently penetrating the BBB, these nanoparticles could minimize side effects commonly associated with systemic drug administration and ensure targeted therapy delivery. Moreover, understanding the safety profile of these engineered nanoparticles is essential, as it addresses concerns related to biocompatibility and potential toxicity. Continuous research into their interaction with both the immune system and neural tissues is imperative to ensure that while they provide effective treatment, they do not provoke adverse reactions within the brain environment [104]. Functionalized surface ligands further enhance target specificity, while biocompatibility ensures prolonged systemic circulation and sustained therapeutic efficacy [105]. This approach represents a transformative strategy to overcome the limitations of conventional drug delivery in neurological disorders. In addition, engineered exosomes with c(RGDyK) peptide efficiently target cerebral ischemic lesions and reduce inflammation, while optimized AAV vectors using directed evolution and receptor modeling enhance BBB transcytosis [106, 107]. They collectively emphasize promising preclinical strategies for targeting central nervous system therapies using innovative delivery systems that cross the BBB.

Heterogeneity within patient populations adds another layer of complexity, as age‐related factors, surgical variations, and individual immune responses can influence therapeutic efficacy. These variations complicate the establishment of standardized treatment protocols and underscore the necessity of personalized therapeutic strategies tailored to patient‐specific risk profiles. Finally, long‐term safety and efficacy remain paramount concerns. Given microglia's essential roles in CNS immune surveillance and homeostasis, prolonged inhibition or dysregulation of microglial activity may have unintended consequences, including increased susceptibility to infections or maladaptive neuroinflammatory responses.

### 6.3 Future Directions

The successful translation of microglia‐targeting therapies into clinically effective treatments for postoperative neurodegenerative diseases (PND) necessitates a multifaceted approach, encompassing advancements in drug delivery, precision medicine, combination therapies, longitudinal clinical trials, and interdisciplinary collaboration [108]. Enhancing BBB permeability remains a critical challenge in optimizing microglial‐targeting therapies. Innovative drug delivery strategies, such as nanoparticle‐mediated transport, intranasal administration, and BBB‐penetrating peptides, offer promising avenues for improving drug bioavailability and CNS penetration. These approaches enable targeted delivery to microglia while minimizing systemic side effects, thereby enhancing therapeutic efficacy.

Precision medicine, informed by biomarker‐guided stratification, holds potential for tailoring treatments to individual genetic, proteomic, or metabolomic profiles. Identifying patient‐specific susceptibilities and underlying PND mechanisms through biomarker analysis can facilitate the development of personalized therapeutic strategies. Key biomarkers, including inflammatory mediators (e.g., IL‐6, TNF‐α), neurotransmitter metabolites (e.g., glutamate, GABA), and genetic markers (e.g., CX3CR1, TREM2), have been identified as predictive indicators of PND progression and treatment responsiveness [97, 109, 110, 111, 112]. Integrating biomarker profiling into clinical decision‐making enables the selection of appropriate microglial modulators, optimization of dosing regimens, and prediction of patient responses to targeted interventions. For instance, individualized cytokine profiling could guide the adjustment of microglial‐targeting agents to mitigate neuroinflammation while minimizing adverse effects. Given the multifactorial nature of PND, combination therapies may be essential for achieving optimal clinical outcomes. Integrating microglial modulators with adjunctive treatments—such as anti‐inflammatory agents, cognitive enhancers, and anesthetic strategies that minimize neurocognitive impact—could provide a more comprehensive approach to PND management.

Despite the limited clinical studies on microglia in PND, research on inflammatory mediators underscores the pivotal role of neuroinflammation in disease progression. For instance, a study on esketamine in elderly patients undergoing gastrointestinal surgery demonstrated a reduction in delayed neurocognitive recovery (dNCR), attributed to attenuated neuroinflammation, as evidenced by decreased serum IL‐6 and S100β levels [113]. Similarly, edaravone (EDA) administration in elderly hip replacement patients improved cognitive function while lowering IL‐6 and CXCL13 levels [114], reinforcing the therapeutic potential of targeting inflammatory pathways to mitigate cognitive decline. Insulin treatment in older elective surgery patients also alleviated postoperative delirium (POD) and reduced IL‐6 and S100β levels [115]. Moreover, probiotics effectively mitigated postoperative cognitive impairment, likely by suppressing peripheral inflammation, as indicated by diminished IL‐6 and cortisol levels [116]. Additionally, the MARBLE study is investigating the perioperative administration of an APOE4 mimetic peptide, CN‐105, in older adults to reduce neuroinflammation and PND severity, targeting APOE4‐related inflammatory pathways implicated in cognitive decline [117]. Another pilot randomized controlled trial demonstrated that a perioperative anti‐inflammatory bundle combining dexmedetomidine, glucocorticoids, ulinastatin, and non‐steroidal anti‐inflammatory drugs (NSAIDs) significantly lowered postoperative delirium incidence in elderly hip fracture patients by reducing systemic inflammation [118]. These findings highlight the critical role of inflammatory modulation in PND management and suggest a complex interplay between systemic inflammation, cognitive outcomes, and therapeutic interventions. Further clinical investigations should elucidate microglial inflammation's contribution to PND pathogenesis.

Interdisciplinary collaboration remains imperative for translating laboratory discoveries into clinical applications. Close integration among neuroscientists, pharmacologists, clinicians, and biotechnologists is essential for designing protocols that seamlessly incorporate microglial‐targeted therapies into standard perioperative care, thereby expediting the transition of these treatments from experimental research to clinical practice.

## 7 Conclusions and Perspectives

Therapeutic modulation of microglia represents a promising strategy for mitigating PND, a prevalent postoperative complication that profoundly affects the quality of life in elderly surgical patients. The preclinical evidence reviewed underscores microglial cells as central mediators of the inflammatory cascade in PND and identifies multiple intervention targets. Strategies such as microglial depletion, phenotype modulation, and the inhibition of specific inflammatory pathways and receptors have demonstrated efficacy in alleviating cognitive dysfunction in animal models. However, translating these findings into clinical applications presents considerable challenges, including the intricate physiology of microglia, the necessity for targeted delivery systems to circumvent the BBB, and the heterogeneity of patient responses. Additionally, the long‐term safety of microglial modulation remains a critical concern given their indispensable role in CNS homeostasis. Future research should prioritize optimizing these therapeutic approaches through advanced drug delivery platforms, leveraging precision medicine to individualize treatments, and conducting extensive, long‐term clinical trials. Furthermore, interdisciplinary collaboration will be essential for overcoming translational barriers and refining treatment protocols. A deeper understanding of microglial dynamics in perioperative care could ultimately pave the way for novel interventions to prevent and manage PND, thereby improving surgical outcomes and patient well‐being.

## Author Contributions

X.L. wrote the main manuscript text and prepared figures. A.Z. prepared the table and figures.

## Ethics Statement

The authors have nothing to report.

## Consent

The authors have nothing to report.

## Conflicts of Interest

The authors declare no conflicts of interest.

## Data Availability

Data sharing not applicable to this article as no datasets were generated or analysed during the current study.
